# Postoperative complications with neuromuscular blocking drugs and/or reversal agents in obstructive sleep apnea patients: a systematic review

**DOI:** 10.1186/s12871-018-0549-x

**Published:** 2018-07-19

**Authors:** Khawaja Rashid Hafeez, Arvind Tuteja, Mandeep Singh, David T. Wong, Mahesh Nagappa, Frances Chung, Jean Wong

**Affiliations:** 1 Department of Anesthesia, Toronto Western Hospital, University Health Network, University of Toronto, 2-405 McLaughlin Wing, 399 Bathurst Street, Toronto, ON M5T 2S8 Canada; 2 Department of Anesthesia, Women’s College Hospital, Toronto, ON Canada; 3 Toronto Sleep and Pulmonary Center, Toronto, ON Canada; 4 Department of Anesthesiology and Perioperative Medicine, University Hospital, St. Joseph’s Hospital and Victoria Hospital, London Health Sciences Centre and St. Joseph’s Health Care, Western University, London, ON Canada

**Keywords:** Obstructive sleep apnea, Postoperative pulmonary complications, Residual neuromuscular blockade, Neuromuscular blocking agents, Reversal of neuromuscular blocking agents

## Abstract

**Background:**

Neuromuscular blocking drugs (NMBD) are administered intra-operatively to facilitate intubation and to achieve muscle relaxation for surgical procedures. Incomplete reversal of NMBD can lead to adverse events in the postoperative period. Patients with obstructive sleep apnea (OSA) may be at higher risk of complications related to the use of NMBD. The objectives of this systematic review were to determine whether: 1) OSA patients are at higher risk of postoperative complications from the use of NMBD than non-OSA patients, and 2) the choice of NMBD reversal agent affects the risk of postoperative complications in OSA patients.

**Methods:**

A literature search of multiple databases was conducted up to April 2017. The inclusion criteria were: (1) adult surgical patients (≥18 years old) with OSA diagnosed by polysomnography, or history, or suspected by screening questionnaire; (2) patients who were given NMBD and/or NMBD reversal agents intraoperatively; (3) reports on postoperative adverse events, particularly respiratory events were available; (4) published studies were in English; and (5) RCTs or observational cohort studies. The quality of evidence was determined by the Oxford Center for Evidence Based Medicine levels of evidence.

**Results:**

Out of 4123 studies, five studies (2 RCTs and 3 observational studies) including 1126 patients were deemed eligible. These studies were heterogeneous precluding a meta-analysis of the results. Two of three studies (1 RCT, 2 observational studies) reported that OSA patients given NMBD may be at higher risk of developing postoperative pulmonary complications (PPCs) like hypoxemia, residual neuromuscular blockade or respiratory failure compared to non-OSA patients. Two studies (1 RCT, 1 observational study) reported that OSA patients who were reversed with sugammadex vs. neostigmine had less PPCs and chest radiographic changes, but the quality of the included studies was Oxford level of evidence: low to moderate.

**Conclusions:**

OSA patients who receive intraoperative NMBD may be at higher risk for postoperative hypoxemia, respiratory failure and residual neuromuscular blockade compared to non-OSA patients. There is some, albeit very limited evidence that NMBD reversal with sugammadex may be associated with less PPCs than neostigmine in patients with OSA. More high-quality studies are needed.

## Background

Obstructive sleep apnea (OSA) is a common form of sleep disordered breathing (SDB). The prevalence has increased over the last two decades in association with the rise of the obesity epidemic [1]. It has been estimated that 13% of men and 6% of women between 30 and 70 years of age have moderate to severe SDB [2]. Its prevalence is even higher in surgical patients [3, 4]. The severity of OSA often worsens after surgery [5] and patients with OSA are at increased risk of postoperative complications, including serious respiratory complications [6–10].

Neuromuscular blocking drugs (NMBD) are widely used intraoperatively to facilitate tracheal intubation and surgical relaxation [11]. These agents act on acetylcholine receptors at the neuromuscular junction to block neurotransmission and hence, decrease airway tone and blunt the protective airway reflexes [12, 13]. However, NMBD have been reported to have residual effects in the postoperative period even after the administration of reversal agents, potentially causing adverse respiratory outcomes [14–16] like decreased inspiratory flow [17], upper airway obstruction, oxygen desaturation, impaired airway protective reflexes, pneumonia, muscle weakness and reintubation in the postoperative period [13, 18, 19]. Residual neuromuscular blockade (NMB) is common and even mild degrees of residual paralysis can have serious clinical consequences [20]. Reversal agents to NMBD are used to antagonize their effects [21]. There are two types of agents; the anticholinesterases (such as neostigmine) and cyclodextrins (sugammadex) [22]. Sugammadex is a newer reversal agent for aminosteroidal NMBD that reverses moderate to deep neuromuscular blockade [22].

OSA patients may be more vulnerable than non-OSA patients to postoperative complications due to NMBD and inadequate reversal of NMBD. The effect of reversal agents on the occurrence of postoperative complications in OSA patients is uncertain, however, sugammadex may have advantages over neostigmine in this patient population.

The purpose of this systematic review is to determine whether: 1) OSA patients who received NMBD as part of general anesthesia may be at higher risk for developing postoperative complications than non-OSA patients who received NMBD; and 2) the choice of NMBD reversal agent affects the risk of postoperative complications in patients with OSA. This review was prepared as part of the Society of Anesthesia and Sleep Medicine’s committee on a guideline for intraoperative management of adult patients with OSA.

## Methods

### Search strategy and study selection

A literature search was performed according to the Preferred Reporting Items for Systematic Reviews guidelines [23]. The search strategy was carried out with the help of a research librarian familiar with literature searches for systematic reviews. We screened published articles describing postoperative complications in surgical patients with OSA and/or obesity who were given NMBD and/ or reversal agents intraoperatively. We included articles that described residual NMB. The literature databases searched were MEDLINE (1946 to April 4, 2017), ePub ahead of print, MEDLINE in-process, and other non-indexed citations (up to April 4, 2017), Embase (1947 to April 4, 2017), Cochrane Central Register of Controlled Trials (up to February, 2017), Cochrane Database of Systematic Reviews (2005 to April 4, 2017), PubMed (1946 to April 4, 2017), Web of Science (1900 to April 4, 2016), Scopus (1960 to April 4, 2017), ClinicalTrials.Gov (up to April 6, 2017), WHO ICTRP (up to April 6, 2017).

The search terms included the Medical Subject Heading keywords “obstructive sleep apnea,” “obesity” and “neuromuscular blockade”. The following text keywords were used for the literature search: “obstructive sleep apnea syndrome,” “sleep disordered breathing,” “obesity hypoventilation syndrome,” “apnea or apnoea,” “hypopnea or hypoapnea,” “muscle relaxant,” “rocuronium,” “atracurium,” “cis-atracurium,” “vecuronium,” “mivacurium,” “suxamethonium or succinylcholine,” “rapacuronium,” “pancuronium,” “skeletal muscle relaxant,” “neuromuscular reversal agents,” “neostigmine,” “edrophonium,” “sugammadex,” “residual neuromuscular block,” “neuromuscular blockade reversal,” “postoperative residual neuromuscular blockade,” “post-extubation complications”, “perioperative complications,” and “postoperative complications.”

Inclusion criteria were: (1) adult surgical patients (≥18 years old) with OSA confirmed by polysomnography (PSG), history, or suspected by screening questionnaire; (2) patients in the study were given NMBD and/ or NMBD reversal agents intraoperatively; (3) reports on postoperative adverse events, particularly respiratory events were available (4) published studies were in English and (5) randomized controlled trials (RCTs) or observational cohort studies. Exclusion criteria were: 1) case reports and review articles; 2) studies with no information on OSA status; and 3) studies with no information on postoperative pulmonary complications (PPCs) and/or residual NMB.

Studies were selected independently by 2 reviewers (RH and AT) who screened the titles and abstracts to determine whether the studies met the eligibility criteria. Disagreements in screening and data extraction were resolved by consulting another author (MS or JW). A citation search by manual review of references from primary or review articles was also performed.

### Data extraction

The following information was collected from each study: author, year of publication, type of study, sample size of OSA and non-OSA group, OSA status (diagnosed or suspected), apnea hypopnea index (AHI), PSG data, and sleep questionnaire data, type of surgery, age, gender, body mass index, NMBD used and dose, NMBD reversal agent used and dose, neuromuscular monitoring, postoperative complications, incidence and type of complication.

PPCs included were airway obstruction, hypoxemia and desaturation, decreased inspiratory capacity, respiratory muscle weakness, residual NMB, increased secretions, bronchospasm, atelectasis, pleural effusion, pulmonary edema, respiratory failure, apnea, respiratory failure, re-intubation, postoperative mechanical ventilation and intensive care unit (ICU) admission. We contacted the authors of the studies for missing data. Only one responded [24].

### Study quality assessment

The risk of bias was evaluated using the Cochrane risk of bias tool [24] for RCTs. For observational cohort studies, the Newcastle-Ottawa tool [25] was used to rate risk of bias and study quality by assessing the selection, comparability and outcome of each study. For evaluation of the quality of evidence, we utilized the Oxford Center for Evidence Based Medicine Levels of Evidence [26].

## Results

### Study selection

**Fig. 1:**
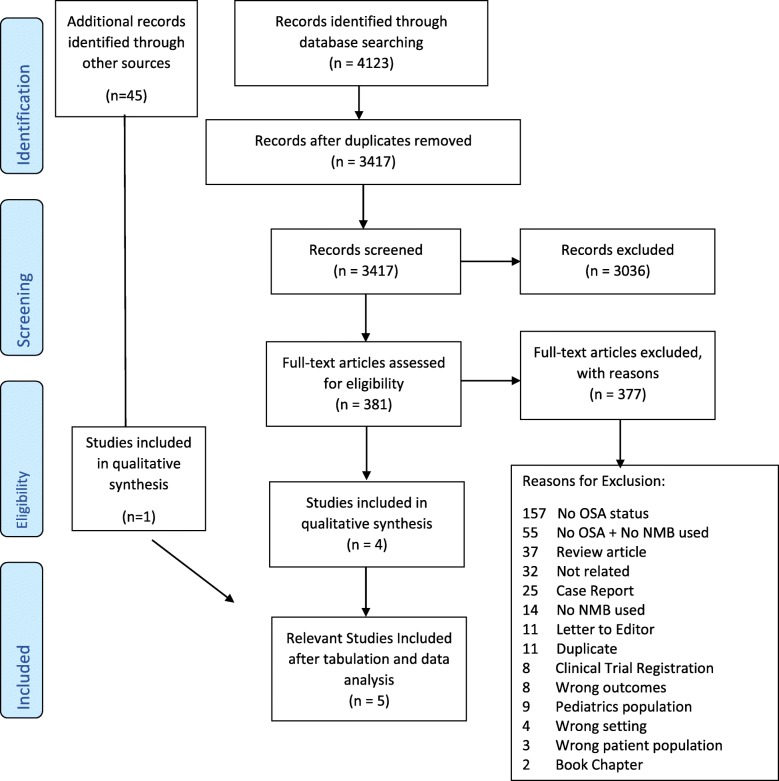
Flow chart of study selection

Our initial electronic search identified 4123 articles, of which four studies were included in the qualitative synthesis (Fig. 1). Forty-five articles were further obtained through citation search and one was included. Three studies describing PPCs in OSA and non-OSA patients were excluded because the number of OSA patients with PPCs was not clearly shown [27–29]. Finally, five studies with at total of 1126 patients were included in this systematic review [30–34].

### Study characteristics

Among the 5 included studies, 2 were RCTs [32, 33] with 426 patients and 3 were observational studies [30, 31, 34] with 700 patients. These were single-center studies from various countries including the United States, Brazil, Turkey and Spain. The types of surgery included were intra-abdominal, bariatric, musculoskeletal, head and neck and otolaryngology surgeries. Descriptive data of the study population are summarized in Table 1. Altogether, 587 diagnosed or suspected OSA patients and 539 non-OSA patients were included. Three studies reported postoperative complications in OSA vs. non-OSA patients who received NMBD [30–32] and 2 reported postoperative complications in OSA patients administered sugammadex vs. neostigmine [33, 34]. One study [30] used the Berlin questionnaire along with preoperative polysomnography to diagnose OSA, and in another study [31], the authors used STOP-BANG criteria for OSA risk assessment. In the other three studies, patients were already diagnosed with OSA. All the included studies used the TOF watch for intraoperative NMB monitoring. In PACU, only Pereira et al. used the TOF Watch for residual NMB monitoring. Unal et al. performed clinical assessment of muscle strength in PACU.

**Table 1:** Descriptive data of study population

Author (year)/ Country	Study design	Age (years) mean ± SDM	Gender (male/female) n	Body mass index (kg/m^2^) mean ± SDM	Total number of subjects	OSA status (n)	Purpose of study	Surgery	Comparators	Oxford LOE
Q 1. OSA vs. non-OSA										
Sudre [32] 2015 Brazil	RCT	IPG 37 ± 11 CG 36 ± 11	IPG 49/151 CG 36/116	IPG 46 ± 6 CG 44 ± 6	352	Already diagnosed OSA (Interventional = 89, Control = 79)	Compared the effects of 2 protocols, Interventional (Remif, Roc, Sevo) vs. Control (Suf, Atracurium, Iso) on immediate recovery time and PPCs after bariatric surgery.	Laparotomy for gastric bypass	Interventional group vs. Control group	2
Ahmad [30] 2009 US	Prospective Cohort	OSA 43 ± 10 Non-OSA 42 ± 12	OSA 8/23 Non-OSA 0/9	OSA 50 ± 9 Non-OSA 48 ± 6	40	Diagnosed OSA (31) by PSG and Berlin questionnaire	To determine whether obese patients with OSA vs. non-OSA were at greater risk for postoperative hypoxemic episodes after bariatric surgery	Laparoscopic bariatric	OSA vs. non-OSA	3
Pereira [31] 2013 Portugal	Prospective Observational	HR-OSA 63 ± 12 LR-OSA 48 ± 18	HR-OSA 113/66 LR-OSA 33/128	HR-OSA 28 ± 5 LR-OSA 24 ± 5	340	STOP-Bang {If ≥3, HR-OSA (179) If 0–2, LR-OSA (161)}	To evaluate the STOP-Bang score in surgical patients in PACU.	Intra-abdominal/bariatric, musculoskeletal, Otolaryngologic	HR-OSA vs. LR-OSA	3
Q 2. Sugammadex vs. Neostigmine										
Unal [33] 2015 Turkey	RCT	Group S (44 ± 9), Group N (46 ± 11)	Not mentioned	Group S 28 ± 3 Group N 28 ± 3	74	Already diagnosed OSA (74)	To compare sugammadex and neostigmine for reversing rocuronium-induced NMB, PPCs s and costs in patients undergoing surgery for treatment of OSA.	Anterior palatoplasty; UPP; tonsillectomy+ UPP, septoplasty; tonsillectomy+ Lateral pharyngoplasty	Sugammadex vs neostigmine	2
Llaurado [34] 2014 Spain	Cohort - Observational	SG 45 ± 35 HG 44 ± 31	SG 32/68 HG 27/73	SG 49 ± 27 HG 46 ± 24	320	Already diagnosed OSA (SG = 74, HG = 61)	To determine whether sugammadex vs. neostigmine to reverse NMB could decrease adverse postoperative respiratory outcomes.	Laparoscopic bariatric	Sugammadex group vs. historical group (neostigmine)	3

*PRCs* postoperative pulmonary complications, *OSA* obstructive sleep apnea, *RCT* randomized control trial, *NMB* neuromuscular block, *PACU* Post-Anesthesia Care Unit, *IPG* interventional protocol group, *CG* conventional group, *UPP* Uvulopalatopharyngoplasty, *HR-OSA* high risk OSA, *LR-OSA* low risk OSA, *Remif* remifentanil, *Roc* 0rocuronium, *Sevo* sevoflurane, *Suf* sufentanil, *Iso* isoflurane, *SG* Sugammadex Group, *HG* historical group, *SD* Standard Deviation, *PSG* Polysomnography

The definitions of postoperative pulmonary complications varied between the different studies (Table 2). Some studies did not define the PPCs or included minor symptoms that are not consistent with previous studies reporting PPCs. Only one study described cardiac complications [33]. The details of postoperative adverse events from each study are shown in Table 3.

**Table 2:** Definitions of postoperative pulmonary complications (PPCs)

Studies	PPCs	Definitions
Sudre [32] (2015)	Atelectasis, pleural effusion, acute pulmonary edema	Chest radiograph findings.
	Respiratory failure	Not defined
Ahmed [30] (2009)	Hypoxemia	SpO_2_ > 4% below preoperative baseline values for > 10 s in duration.
Pereira [31] (2013)	Hypoxemia	Mild-moderate hypoxia (SpO_2_ of 93–90%) on 3 L nasal cannula O_2,_ not improved after active interventions (increasing O_2_ flows to > 3 L/min, application of high-flow face mask O_2_, verbal requests to breathe deeply and tactile stimulation); Severe hypoxia (SpO2 < 90%) on 3 L nasal cannula O2 not improved after active interventions (increasing O_2_ flows to > 3 L/min, application of high-flow facemask O2, verbal requests to breathe deeply, and tactile stimulation).
	Respiratory failure	Signs of respiratory distress or impending ventilatory failure (respiratory rate > 20 breaths per minute, accessory muscle use, and tracheal tug).
	Airway obstruction	Patient complaining of symptoms of respiratory or upper airway muscle weakness (difficulty breathing, swallowing, or speaking), requiring reintubation in the PACU.
	Residual NMB	TOFR < 0.9 and was quantified at PACU admission using acceleromyography of the adductor pollicis muscle (TOF-Watch®).
	Others (airway obstruction, muscle weakness, decreased inspiratory capacity, bronchospasm)	Not defined.
Unal [33] (2015)	Hypoxemia	SpO_2_ ≤ 90% in PACU.
	Airway obstruction	Requiring an intervention (jaw thrust, oral or nasal airway, intubation).
	Apnea	Not defined.
	Re-intubation & invasive postoperative mechanical ventilation.	Patient whose hypoxemia and airway obstruction did not improve despite the application of oxygen through a mask and airway maneuvers.
Llaurado [34] (2014)	Atelectasis, pleural effusion	Chest radiograph finding.

*PPCs* postoperative pulmonary complications, *SpO*_*2*_ Oxygen saturation, *O*_*2*_ Oxygen, *PACU* Post anesthesia care unit, *NMB* neuromuscular blockade, *TOFR* train of four ratio

**Table 3:** Details of postoperative pulmonary complications

Study	NMBD used	NMBD dose	Reversal Used	Reversal Dose	NMBD monitoring (TOFR)	Postoperative complications	Conclusion
Q 1. OSA vs. non-OSA							
Sudre [32] 2015	Succinylcholine + Rocuronium or Atracurium	Induction: succinylcholine 1 mg.kg^− 1^ TBW & interventional group (rocuronium 0.1 mg.kg^− 1^.hr.^− 1^ IBW) Control group (atracurium 0.4 mg.kg^− 1^) Maintenance: IPG (rocuronium 0.1 mg.kg-1.hr.^− 1^IBW) CG (atracurium 0.04 mg.kg^− 1^)	Not specified	Not specified	Yes	OSA was associated with a higher risk of postoperative respiratory failure. (OR 6.88) No difference in atelectasis, bronchospasm, pleural effusion, pulmonary edema	Higher risk of postoperative respiratory failure in OSA vs. non-OSA patients receiving NMBA.
Ahmad [30] 2008	Succinycholine + rocuronium	Succinylcholine (0.5 mg/kg), rocuronium (0.5 mg/kg)	Neostigmine + glycopyrrolate	Neostigmine 0.05 mg.kg^− 1^ (IBW) + Glycopyrolate 0.005 mg.kg^− 1^ (IBW)	Yes	No difference in median SpO_2_ in OSA vs. non-OSA patients given supplemental oxygen in first 24 h after surgery, *P* = 0.97	OSA did not increase the risk for postoperative hypoxemia
Pereira [31] 2013	NMB - drug not specified	Not specified	Neostigmine - where required	Not specified	Yes	Postoperative mild/moderate hypoxia was higher in HR-OSA vs. LR-OSA patients (*n* = 15 vs.4, *P* = 0.012). Significantly higher residual NMB was found in HR-OSA vs. LR-OSA patients (*n* = 35 vs.25, *P* = 0.035).	Mild/moderate hypoxemia (*P* = 0.012) and residual neuromuscular blockade are more frequent in HR-OSA patients vs. LR-OSA patients (*P* = 0.035).
Q 2. Sugammadex vs. Neostigmine							
Unal [33] 2015	Rocuronium	Total rocuronium dose group S = 2.6 + − 16.7 mg, group *N* = 85.0 + − 14.7 mg.	Sugammadex / neostigmine	SG: 2 mg.kg^− 1^ sugammadex, *n* = 37 and 0.04 mg.kg^− 1^ N: neostigmine+ 0.02 mg.kg^− 1^ atropine. *n* = 37.	Yes	PPCs (desaturation, hypoxemia, apnea, airway manipulation, airway usage, re-intubation, CPAP, invasive mechanical ventilation) were lower in OSA patients reversed with sugammadex vs neostigmine, *P* = 0.048. Cardiovascular complications (bradycardia) lower with sugammadex vs. neostigmine, *P* = 0.04.	Postoperative pulmonary complications and bradycardia occurred less frequently in OSA patients who received sugammadex vs. neostigmine (*P* < 0.05).
Llaurado [34] 2014	Succinycholine/ Rocuronium/ Cis-atracurium	SG: succinylcholine 1 mg.kg^− 1^ RBW or rocuronium 1 mg.kg^− 1^ IBW + rocuronium 0.15 mg.kg^− 1^ at T2. HG: succinylcholine 1 mg.kg^− 1^ RBW or rocuronium1 mg.kg^− 1^ IBW or cis-atracurium 0.2 mg/ kg IBW + cis-atracurium 0.03 mg.kg^− 1^	Sugammadex / neostigmine	SG: sugammadex 4 mg.kg^− 1^ + 2 mg.kg^− 1^ (TOFR< 0.9, 3 min apart); HG: neostigmine 0.04 mg.kg^− 1^ + 0.02 mg.kg^− 1^	Yes	Significantly less postoperative abnormalities on chest radiograph (atelectasis, pleural effusions) were observed in the SG: 6.9% (*n* = 11) vs. HG 16.3% (*n* = 26). *P* = 0.015 No difference in need for mechanical ventilation in SG vs. HG (2 vs.5), *P* = 0.38 or hospital stay (3 vs.4, *P* = 0.3).	Significantly less postoperative chest radiograph changes in the OSA patients receiving sugammadex vs. neostigmine. No difference in postoperative mechanical ventilation, and hospital stay.

*RBW* real body weight, *IBW* ideal body weight, *RNMB* residual neuromuscular blockade, *HR-OSA* high risk OSA, *LR-OSA* low risk OSA, *SG* Sugammadex Group, *HG* historical group, *TOFR* train of four ratio, *PTC* post tetanic count, *SO* super-morbidly obese, *MO* morbidly obese, *PPCs* postoperative pulmonary complications, *PACU* Post-Anesthesia Care Unit, *AHI* apne-hyponea index, *Group S* sugammadex, *Group N* neostigmine, *CPAP* continuous positive airway pressure, *DB* deep block, *MB* moderate block, *PACU* postoperative anesthesia care unit

### Risk of Bias

The risk of bias in both RCTs was moderate overall (Table 4). The risk of bias was fair in the observational studies by Ahmed et al. [30] and Llaurado et al. [34] and that of Pereira et al. [31] was good (Table 5). The quality of all studies was moderate to low (Table 1).

**Table 4:** Cochrane Risk of Bias in Included Studies

First author (yr)	Adequate sequence generation	Allocation concealment	Blinding	Blinding of outcome assessment	Incomplete outcome data assessed	Free of selective outcome reporting	Free of other biases
Unal (2015) [33]	Unclear	Unclear	Unclear	Unclear	Yes	Yes	Yes
Sudre (2015) [32]	Unclear	Unclear	Unclear	Yes	Yes	Yes	Unclear

**Table 5:** Newcastle-Ottawa Scale

Quality of Included Studies Assessed by Using the Newcastle-Ottawa Quality Scale for Comparative Studies																											
Study	Study design	Selection (Max = 4 stars)													Comparability (Max = 2Stars)		Outcome (Max = 3Stars)										Total
		1				2			3				4		1		1				2		3				
		A*	B*	C	D	A*	B	C	A*	B*	C	D	A*	B	A*	B*	A*	B*	C	D	A*	B	A*	B*	C	D	
Ahmed [30] 2009	Prospective Cohort					*			*				*								*		*				5
Pereira [31] 2013	Prospective Cohort		*			*				*			*					*			*		*				7
Llaurado [34] 2014	Prospective Cohort					*							*		*			*			*		*				6

*Questions marked with asterisk that are fulfilled will award the study one star; fulfillment of non-asterisked columns awards no stars*

***Selection***

1) Representativeness of the exposed cohort:

A) truly representative of the average population; B) somewhat representative of the average population; C) selected group of users; D) no description of the derivation of the cohort

2) Selection of the non-exposed cohort:

A) drawn from the same community as the exposed cohort; B) drawn from a different source; C) no description of the derivation of the non-exposed cohort;

3) Ascertainment of exposure:

A) secure record; B) structured interview; C) written self-report; D) no description

4) Demonstration that outcome of interest was not present at start of study:

A) yes; B) no

***Comparability***

1) Comparability of cohorts on the basis of the design or analysis:

A) study controls for cohort__; B) study controls for any additional factor

***Outcome***

1) Assessment of outcome:

A) independent blind assessment; B) record linkage; C) self-report; D) no description

2) Was follow-up long enough for outcomes to occur

A) yes (select an adequate follow up period for outcome of interest); B) no

3) Adequacy of follow up of cohorts

A) complete follow up - all subjects accounted for; B) subjects lost to follow up unlikely to introduce bias; C) follow up rate is adequate and no description of those lost; D) no statement

Scoring algorithm*

**Table:** 

Quality rating	# Points in Selection Domain	# Points in Comparability Domain	# Points in Outcome Domain
Good	≥3	≥2	≥2
Fair	2	≥1	≥2
Poor	0-1	0	0-1

Are patients with OSA who received NMBD as part of general anesthesia at higher risk for postoperative complications than patients without OSA?

We identified one RCT [32] and 2 observational studies [30, 31] with 378 diagnosed or suspected OSA patients and 345 non-OSA patients. Since the studies were heterogeneous and differed in study design, types of surgery, and outcomes reported, a meta-analysis was not performed.

In a RCT of 352 patients, Sudre et al. found that OSA vs. non-OSA patients were at higher risk of developing respiratory failure (OR 6.88, 95% CI 2.36–20.05, *P* = 0.0004) [32]. In an observational study of 40 patients, Ahmed et al. reported that OSA was not an independent risk for postoperative hypoxemia in the first 24 h after laparoscopic bariatric surgery (*P* = 0.97) [30]. In another observational study of 340 patients, Pereira et al. [31] showed that patients with high risk of OSA (STOP-Bang score ≥ 3) vs. low risk patients (STOP-Bang 0–2) more frequently experienced residual NMB (24% vs. 17%, *P* = 0.035), and mild/moderate hypoxia (9% vs. 3%, *P* = 0.012). There was no significant difference in other respiratory complications between OSA vs. non-OSA patients who received NMBD [30–32]. Based on the included studies, OSA vs. non-OSA patients who have received NMBD may be at higher risk of hypoxemia, residual NMB and respiratory failure (Oxford LOE between 2 and 3) (Table 1).

Does the choice of NMBD reversal agent affect the risk of postoperative complications in OSA patients?

We identified one RCT [33] and one observational study [34] with a total of 206 OSA patients that evaluated the impact of reversal agents on postoperative complications (Table 2). Two reversal agents, sugammadex and neostigmine, were compared.

In the RCT of 74 OSA patients, Unal et al. found that sugammadex decreases the incidence of PPCs {desaturation, hypoxemia, apnea, airway manipulation, airway usage, re-intubation, CPAP (continuous positive airway pressure), invasive mechanical ventilation (Table 3)}. Eight patients (21.6%) had significant bradycardia (*P* = 0.028) with six requiring treatment with atropine [33]. In an observational study of 145 OSA patients undergoing laparoscopic bariatric surgeries, sugammadex was found to decrease the incidence of postoperative chest radiograph changes {atelectasis, pleural effusions (*P* = 0.007)} compared with a historical cohort receiving neostigmine. No difference in clinical outcomes including postoperative mechanical ventilation or hospital stay was found [34]. Oxford LOE for both studies was between low to moderate (Table 1).

## Discussion

To date, this systematic review is the first to examine the association of NMBD with PPCs in OSA vs. non-OSA patients, and the impact of NMBD reversal agents on PPCs in patients with OSA. Though the evidence is limited, our systematic review suggests that OSA patients who are given NMBD may be at a higher risk for hypoxemia, residual NMB and respiratory failure than non-OSA patients. As well, 2 studies reported that OSA patients who received sugammadex have less postoperative complications than those receiving neostigmine, however the studies were of low quality.

### OSA vs. non-OSA

There is growing concern that residual NMB is associated with adverse respiratory outcomes in patients undergoing anesthesia [22, 35–38]. Residual NMB is particularly important to avoid in OSA patients since OSA is associated with an increased risk of postoperative respiratory and cardiac events, ICU transfers, and longer hospital stay [6–8, 39, 40]. Anatomical risk factors increase vulnerability to airway collapse during sedation or sleep in OSA patients. The retropalatal, retroglossal and hypopharyngeal regions of the upper airway in OSA patients are the most common sites of collapse during sleep or sedation, causing obstruction [41]. This collapse can occur because of variations in transmural pressure, such as decreased intraluminal pressure or increased external tissue pressure, or a reduction in the longitudinal tension on the pharynx [42]. Magnetic resonance imaging (MRI) studies show that the airway in OSA patients is different from the normal airway because of thicker lateral pharyngeal walls with an anteroposterior elliptical configuration, unlike the horizontal configuration in the normal airway leading to airway narrowing [42].

Upper airway dilators are more vulnerable to the effects of NMBD compared to the respiratory pump muscles [6, 15]. Even at levels producing mild blockade, as measured by train of four ratio (TOFR) 0.7–.9, NMBD increased upper airway collapsibility and impaired compensatory genioglossus response to negative pharyngeal pressure challenges [43]. Due to the pathophysiology of the disease, patients with OSA may have increased vulnerability to the effects of NMBD and reversal agents [42, 43]. In a large retrospective database study of 530,089 patients with 32,789 diagnosed OSA patients, Memtsoudis et al. [8] found a 5-fold increase in intubation and mechanical ventilation in OSA patients after orthopedic surgery and a 2-fold increase after general surgery compared with non-OSA patients. They concluded that OSA is an independent risk factor for developing pulmonary complications [8].

In our review, two observational studies found contrary results for hypoxemia [30, 31]. Ahmed et al. found that OSA patients were not at higher risk of developing hypoxemia than non-OSA patients. A significant limitation is the small sample size (*n* = 41; OSA = 31, non-OSA =9). Also, the use of supplemental oxygen at 3 l per min via nasal cannula for 24 h postoperatively in all patients may have masked the occurrence of desaturation episodes. The incidence of hypoxemia was significantly higher in morbidly obese patients with or without OSA, suggesting the importance of morbid obesity as an independent risk factor for PPCs [30]. Pereira et al. concluded that mild/moderate hypoxia was the only PPC in the immediate postoperative period that occurred more frequently in patients with suspected OSA [31]. Schumann et al. studied the relation between metabolic syndrome and surgical factors (duration and type of surgery) with PPCs in bariatric patients [44]. They found that increasing age, BMI, ASA status, metabolic syndrome, OSA, asthma, congestive heart failure, surgical factors were independently associated with PPCs. PPCs and metabolic syndrome were significantly associated with increased postoperative mortality [42]. Many other studies have also reported that OSA patients are at a higher risk for hypoxemia than non-OSA patients [5, 35–38].

### Monitoring of NMB

Residual NMB was monitored in all the included studies by TOFR using acceleromyography before reversal agent, and on admission in the PACU [30–32]. In the general population, residual NMB increases the incidence and risk of PPCs in a dose dependent manner [16]. To avoid PPCs, neuromuscular monitoring is important to reduce residual NMB; this is even more important in OSA patients as it has been reported as an associated risk factor for early PPCs requiring invasive airway placement or intensive respiratory care [7, 35]. Ideally, before extubation, any residual NMB should be ruled out by quantitative neuromuscular monitoring in a train of four (TOF) supramaximal peripheral nerve stimulations administered over two seconds [45]. A consensus statement on the perioperative use of neuromuscular monitoring was recently published by a panel of clinician scientists with expertise in NMB monitoring [46].^.^ They recommended that whenever a NMBD is administered, neuromuscular function must be monitored by observing the evoked muscular response to peripheral nerve stimulation [46]. The American Society of Anesthesiologists recommends that: 1) patients at increased perioperative risk from OSA should be extubated while awake; 2) full reversal of NMB should be verified before extubation; and 3) when possible, extubation and recovery should be carried out in the lateral, semi-upright, or other non-supine positions [47].

### Use of reversal agents

Several studies have examined sugammadex vs. neostigmine in patients without OSA. Brueckmann et al. and Sabo et al. found that the use of sugammadex vs. neostigmine for NMBD reversal reduced residual NMB in PACU [48, 49]. In a systematic review of 17 RCTs of non-OSA patients, Abad-Gurumeta et al. found a significant reduction in residual NMB with sugammadex vs. neostigmine but no difference in the rate of adverse respiratory events that required tracheal re-intubation [50]. We found some, albeit limited evidence to support a reduction in PPCs in OSA patients receiving sugammadex vs. neostigmine. In a 2017 Cochrane review, significantly less bradycardia occurred in patients who received sugammadex vs. neostigmine [36]. In the OSA patients, Unal et al. also reported less bradycardia in the sugammadex vs. neostigmine group [33].

In the morbidly obese patients, Gaszynski et al. found that sugammadex reversed rocuronium more quickly than neostigmine with a mean time to 90% of TOF (2.7 vs. 9.6 min, *P* < 0.05), and a higher TOF at the PACU (109.8% vs 85.5%, *P* < 0.05) [51]. In the obese vs. patients with a normal body mass index, the duration of action of NMBD may be prolonged which may lead to increased incidence of residual NMB in PACU [52]. In both studies, PPCs and the number of OSA patients were not described [51, 52]. In contrast, in a review of 27 studies with over 1400 patients receiving sugammadex, Monk et al. reported that there were no clinically significant differences in recovery times to a TOFR of 0.9 between patients with and without obesity, following reversal of NMBD with sugammadex [53].

The Society of Anesthesia and Sleep Medicine recommended that OSA patients should be identified and optimized preoperatively and the diagnosed OSA patients who are already on home CPAP device should continue with CPAP perioperatively [54]. These precautions should be taken when NMBD are administered for OSA patients.

### Limitations

There were few studies evaluating the effect of NMBD on postoperative complications in OSA vs. non-OSA patients, and the effect of different reversal agents on postoperative complications in patients with OSA. The sample size of included studies was small. There was a lack of consistent definitions for PPCs among the different studies, and some studies reported symptoms such as cough or breath-holding which are not accepted definitions of PPCs [55]. Most studies used rocuronium in standard intubation and maintenance doses, however, benzylisoquinolines were used by Llaurado et al. (cisatracurium) and Sudre et al. (atracurium) only in the control group to compare with rocuronium. In addition, the effect of opioids and residual anesthetics on postoperative pulmonary complications was not evaluated in the studies. OSA patients with co-existing morbidities such as chronic obstructive pulmonary disease, renal or liver disease were not evaluated. As OSA is associated with significant comorbidities, such as morbid obesity, obesity hypoventilation syndrome, pulmonary hypertension and cardiovascular diseases, it is unclear what the contribution of OSA or its comorbidities are towards PPCs [52]. Only Pereira et al. measured TOF in the PACU. Finally, one study used the STOP-Bang questionnaire to identify high risk OSA and the diagnosis was not confirmed by polysomnography [31].

## Conclusions

Our systematic review suggests that OSA patients who received intraoperative NMBD may be at higher risk for postoperative residual neuromuscular blockade, hypoxemia, and respiratory failure. The use of sugammadex was associated with less postoperative pulmonary complications in patients with OSA as compared to neostigmine, however, the evidence was very limited as the studies were of low to moderate quality. Larger, well-designed trials are needed to elucidate the effect of sugammadex on postoperative complications in OSA patients.

## Abbreviations

AHI: Apnea-hypopnea index
CPAP: Continuous positive airway pressure
ICU: Intensive care unit
NMB: Neuromuscular blockade
NMBD: Neuromuscular blocking drugs
OSA: Obstructive sleep apnea
PACU: Post anesthesia care unit
PPCs: postoperative pulmonary complications
PSG: Polysomnography
RCT: Randomised controlled trial
SDB: Sleep disordered breathing
TOFR: Train of four ratio
